# Low-Grade Trichoblastic Carcinoma of the Scalp: A Rare Case Managed With Adjuvant Radiotherapy

**DOI:** 10.7759/cureus.109388

**Published:** 2026-05-21

**Authors:** Jorge A Valdivieso, Rebecca K Hicks

**Affiliations:** 1 Internal Medicine-Pediatrics, Marshall University Joan C. Edwards School of Medicine, Huntington, USA; 2 Medicine, Marshall University Joan C. Edwards School of Medicine, Huntington, USA

**Keywords:** adjuvant radiotherapy, adnexal skin tumor, basal cell carcinoma mimic, scalp neoplasm, trichoblastic carcinoma

## Abstract

Trichoblastic carcinoma (TBC) is a rare malignant adnexal tumor originating from follicular germinative cells. It can resemble basal cell carcinoma (BCC) both clinically and histologically, often leading to diagnostic and therapeutic challenges.

We report a 44-year-old male patient who presented with a recurrent exophytic scalp lesion initially diagnosed as pilomatrixoma. Following wide local excision, histopathologic analysis revealed a low-grade TBC with narrow margins. Due to the close proximity of tumor cells to the surgical margins, the patient underwent adjuvant volumetric modulated arc therapy (VMAT) to a total dose of 50 Gy in 20 fractions.

This case underscores the importance of accurate histopathologic differentiation between TBC and BCC. Surgical excision with adequate margins remains the treatment of choice, while adjuvant radiotherapy may be warranted in cases with narrow or positive margins to prevent recurrence.

## Introduction

Trichoblastic carcinoma (TBC) is an uncommon malignant adnexal neoplasm derived from follicular germinative cells. It is considered the malignant counterpart of trichoblastoma and represents less than 1% of all cutaneous adnexal carcinomas [1]. Clinically and histopathologically, TBC closely mimics basal cell carcinoma (BCC), making accurate diagnosis essential, as management strategies and biologic behavior may differ significantly between the two entities [2].

While most TBCs are indolent, some exhibit locally aggressive behavior or potential for metastasis, particularly when incompletely excised [3]. Optimal management typically involves wide surgical excision with histologically clear margins. However, the role of adjuvant radiotherapy remains poorly defined and is generally reserved for cases with positive or close margins, perineural invasion, or unresectable disease [4]. We present a case of low-grade TBC of the interparietal scalp in a 44-year-old man, managed with wide local excision followed by adjuvant radiotherapy due to narrow surgical margins.

## Case presentation

A 44-year-old man with a medical history of epilepsy, managed with carbamazepine (Tegretol) for several years, presented with a slowly enlarging lesion on the interparietal scalp. The patient reported that one year earlier, the lesion had been excised at another institution, with histopathology consistent with pilomatrixoma and positive surgical margins. The lesion recurred within months and continued to enlarge gradually.

**Figure 1:**
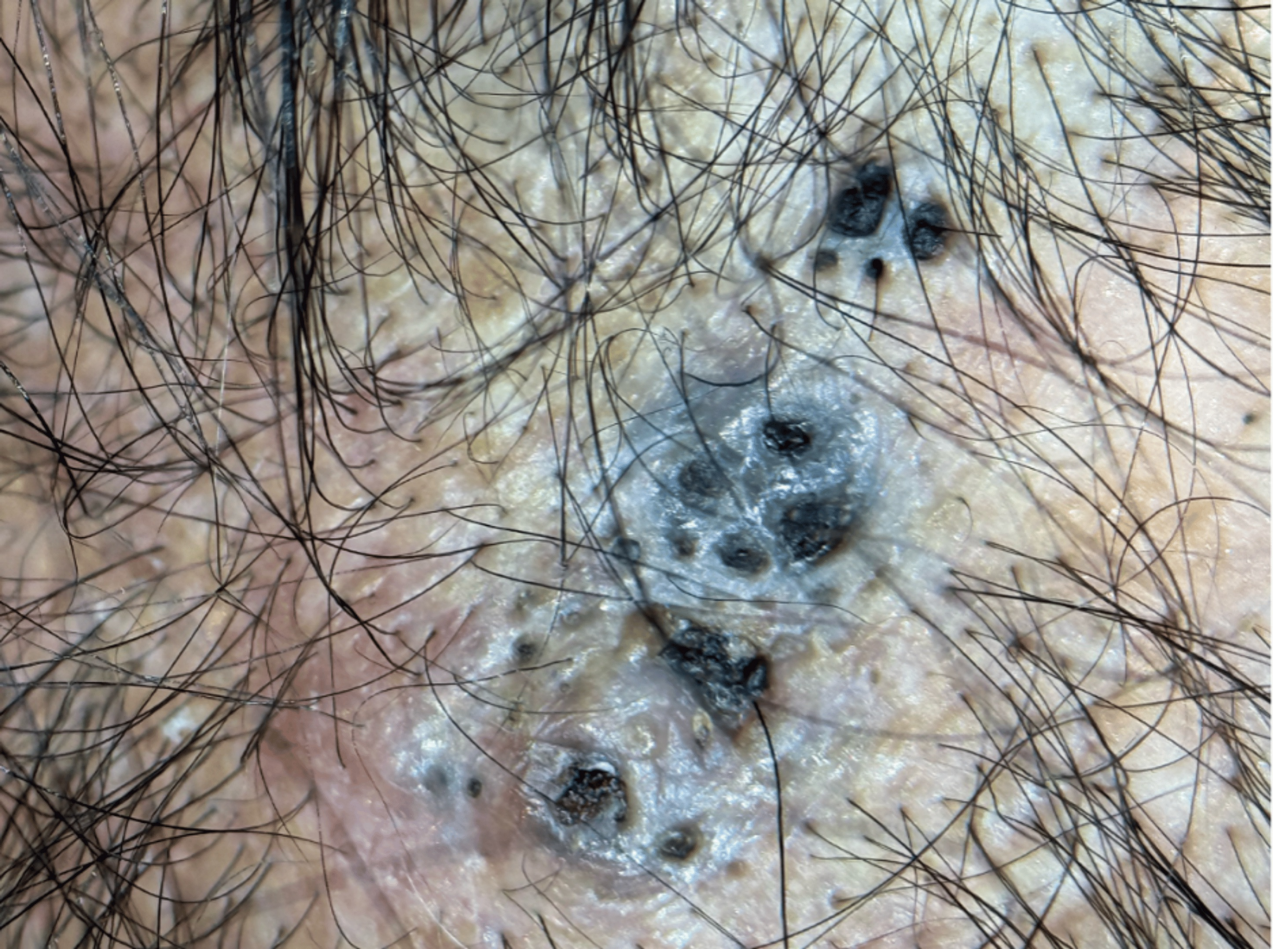
Exophytic scalp lesion with keratin-filled openings

On examination, a 3 cm hyperpigmented, exophytic mass with multiple keratin-filled openings and moderate serous discharge was observed on the interparietal scalp (Figure 1). There was no regional lymphadenopathy or systemic symptoms.

The patient underwent a wide local excision under general anesthesia. Gross examination revealed a lobulated dermal tumor with irregular borders.

**Figure 2:**
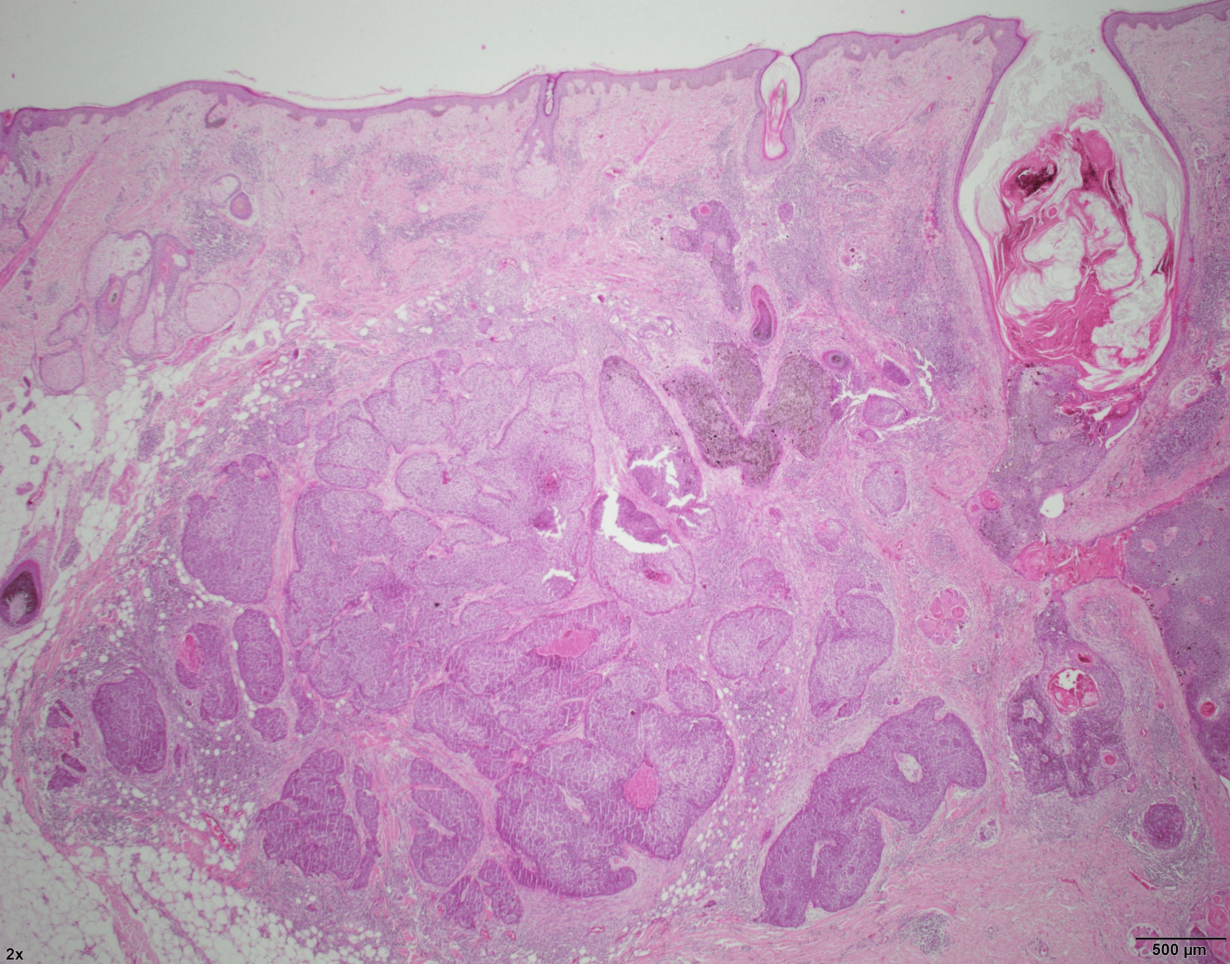
Histopathologic features of low-grade trichoblastic carcinoma

Histopathologic analysis demonstrated basaloid cell proliferation with follicular differentiation, peripheral palisading, fibrous stroma, and associated dendritic melanocytic colonization without significant cytologic atypia or necrosis (Figure 2). The surgical margins were narrow (<1 mm) but free of tumor.

Given the close margins and the potential for local recurrence, the multidisciplinary tumor board recommended adjuvant radiotherapy. The patient received 50 Gy in 20 fractions using volumetric modulated arc therapy (VMAT) to the tumor bed and 1 cm margin of surrounding tissue.

Treatment was well-tolerated with only mild radiation dermatitis. At the six-month follow-up, the patient demonstrated a well-healed surgical site with no evidence of local or regional recurrence.

## Discussion

TBC encompasses a spectrum of follicular germinative cell neoplasms that range from indolent, low-grade tumors to aggressive, high-grade variants [5]. Low-grade TBC typically follows an indolent clinical course but retains the potential for local recurrence, particularly in cases of incomplete excision [6].

Histologically, distinguishing TBC from BCC remains a diagnostic challenge. Both tumors exhibit basaloid proliferation and peripheral palisading, but TBC often demonstrates more prominent follicular differentiation and fibrous stroma, while often lacking the characteristic mucinous stroma and stromal retraction commonly seen in BCC [7]. Immunohistochemistry may aid in distinguishing TBC from BCC. Trichoblastic tumors may demonstrate colonization by CK20-positive Merkel cells and expression of follicular stem cell markers such as PHLDA1, while BCC typically demonstrates diffuse Ber-EP4 positivity and lacks Merkel cell colonization [8]. Given the rarity and overlapping histopathologic features of follicular neoplasms, definitive classification may remain challenging in select cases despite expert dermatopathologic review.

In this case, initial misdiagnosis as pilomatrixoma likely resulted from overlapping histopathologic features, emphasizing the need for careful diagnostic review of recurrent or atypical adnexal lesions. The differential diagnosis also included melanocytic matricoma, given the prominent dendritic melanocytic proliferation and matrical differentiation. However, the absence of prominent shadow cells, overt matrical differentiation, and high-grade cytologic atypia were overall favored to represent low-grade TBC. Immunohistochemical evaluation, including melanocytic markers, was reviewed during pathologic assessment to exclude a concomitant melanocytic neoplasm. Additionally, prior biopsy slides were re-reviewed alongside the excision specimen during multidisciplinary pathologic evaluation to ensure diagnostic concordance.

The mainstay of treatment is complete surgical excision with histologically negative margins. Mohs micrographic surgery may be preferred for facial or scalp lesions because it allows complete margin assessment while maximizing tissue preservation in cosmetically and functionally sensitive areas [9]. In our case, although margins were free of tumor, they were extremely narrow (<1 mm), prompting consideration of adjuvant therapy.

Adjuvant radiotherapy for TBC is not standard but can be beneficial when margins are close or positive, when re-excision would cause significant morbidity, or in recurrent disease [10]. VMAT provides excellent conformality and tissue sparing, allowing high-dose delivery to the target while minimizing exposure to surrounding scalp and brain tissue. Immobilization was achieved using a custom thermoplastic head mask to ensure reproducible positioning during treatment delivery. A tissue-equivalent bolus was utilized to optimize surface dose coverage to the scalp. Dose planning included evaluation of isodose distribution and dose-volume histograms to maximize target coverage while minimizing exposure to adjacent normal brain tissue. Although electron beam therapy is commonly utilized for superficial cutaneous malignancies because of its favorable dose fall-off characteristics, VMAT was selected in this case due to the irregular contour of the scalp surgical bed and the need for highly conformal coverage adjacent to critical underlying structures. VMAT allowed improved dose homogeneity and sparing of uninvolved scalp and intracranial tissues. Other radiotherapeutic modalities used in cutaneous oncology include electron beam therapy and brachytherapy. Electron therapy is often preferred for superficial lesions because of limited deep tissue penetration, while brachytherapy may provide highly localized treatment for select nonmelanoma skin cancers. However, data regarding their use specifically in TBC remain limited due to the rarity of the disease.

Our patient achieved excellent local control at the six-month follow-up with no evidence of recurrence, suggesting that adjuvant radiotherapy may serve as a useful adjunct in select cases of incompletely excised or high-risk TBC. The patient experienced mild transient alopecia within the treatment field without evidence of permanent alopecia at the six-month follow-up.

## Conclusions

TBC is a rare adnexal malignancy that can mimic basal cell carcinoma, often leading to diagnostic delays. Accurate histopathologic differentiation is critical for appropriate management. While wide excision remains the cornerstone of therapy, adjuvant radiotherapy should be considered for cases with narrow or positive margins. This case adds to the limited literature supporting the use of radiotherapy as an effective adjuvant modality for local control in TBC.
